# An insight into the genome-wide analysis of bacterial defense mechanisms in a uropathogenic *Morganella morganii* isolate from Bangladesh

**DOI:** 10.1371/journal.pone.0313141

**Published:** 2025-01-23

**Authors:** Syed Muktadir Al Sium, Barna Goswami, Sanjana Fatema Chowdhury, Showti Raheel Naser, Mihir Kanti Sarkar, Md. Jobaid Faruq, Md. Ahashan Habib, Shahina Akter, Tanjina Akhtar Banu, Md. Murshed Hasan Sarkar, Md. Salim Khan

**Affiliations:** 1 Bangladesh Council of Scientific and Industrial Research (BCSIR), Dhaka, Bangladesh; 2 Department of Microbiology, Primeasia University, Dhaka, Bangladesh; Tribhuvan University, NEPAL

## Abstract

The gram-negative, facultative anaerobic bacterium *Morganella morganii* is linked to a number of illnesses, including nosocomial infections and urinary tract infections (UTIs). A clinical isolate from a UTI patient in Bangladesh was subjected to high-throughput whole genome sequencing and extensive bioinformatics analysis in order to gather knowledge about the genomic basis of bacterial defenses and pathogenicity in *M*. *morganii*. With an average nucleotide identity (ANI) of more than 97% similarity to a reference genome and phylogenetic analysis verified the isolate as *M*. *morganii*. Genome annotation identified 3,718 protein-coding sequences, including genes for metabolism, protein processing, stress response, energy, and membrane transport. The presence of biosynthetic gene clusters points to the isolate’s ability to create bioactive compounds, including antibiotics. Genomic islands contained genes for metal transporters, stress proteins, toxin proteins, and genes related to horizontal gene transfer. The beta-lactam resistance gene blaDHA was found using antimicrobial resistance (AMR) gene analysis across three databases. The virulence genes kdsA and cheY, which may be involved in chemotaxis and lipopolysaccharide production, were also available in the isolate, suggesting its high pathogenicity. The genome contained mobile genetic components and defense mechanisms, such as restriction modification and CRISPR-Cas systems, indicating the bacterium’s ability to defend itself against viral attacks. This thorough investigation sheds important light on *M*. *morganii*’s pathogenicity and adaptive tactics by revealing its genetic characteristics, AMR, virulence components, and defense mechanisms. For the development of targeted treatments and preventing the onset of resistance in clinical care, it is essential to comprehend these genetic fingerprints.

## Introduction

*Morganella morganii*, rod shaped gram-negative facultative anaerobic bacteria which was first isolated from a pediatric faecal culture [1]. The genome size of *M*. *morganii* is about 4Mb, and it contains about 4000 protein coding sequences (CDSs) [2]. *M*. *morganii* is widely available in the environment and in some of the animals intestinal tract as part of the normal flora [3].

*M*. *morganii* is considered an uncommon cause of community-acquired infection, and it is most often encountered in postoperative and other nosocomial infections, such as urinary tract infections (UTI). About 60% of women experience urinary tract infections at least once in their lifetime [2, 4, 5]. Patients with long-term urinary catheters are frequently infected with *M*. *morganii* [6]. Around 405 million people are anticipated to be affected by UTIs globally and approximately 0.23 million UTI-related deaths occurred in 2019 [7]. Bangladesh is also facing the disease burden with high number of mortality and economic loss due to longer treatment periods. Recent studies suggest an increasing prevalence of *M*. *morganii* in UTIs. A large survey conducted in Central European Urology Departments (2011–2019) revealed that *M*. *morganii* accounted for a significant portion (6.2%) of positive pathogens identified in urine cultures [8]. This trend is further supported by another research. *Morganella morganii* shared 1.1% of the pathogens causing urinary tract infections in the Asia-Pacific region [9].

Bacteria have encountered a variety of threats from other microbes throughout their evolutionary history, including biotic and abiotic threats [10–12]. In response to these vulnerabilities, bacteria developed complex defense systems that protect them from natural stress, antibiotics and other medicines. These defense mechanisms show insights into the evolutionary perspectives and the changes or mechanisms they used to survive in such conditions. These defensive responses of bacteria [13–15] can increase virulence and protect against antimicrobial treatment, thereby exacerbating disease [16, 17]. The ability of *M*. *morganii* to produce virulence factors such as urease, hemolysins, and lipopolysaccharide (LPS) makes it an opportunistic pathogen that primarily causes wound and urinary tract infections [18]. *Morganella morganii* has intrinsic resistance to ampicillin, amoxicillin and most of the first- and second-generation cephalosporins because of its intrinsic AmpC resistance gene [19]. The continued acquisition of resistance genes or virulence factors through mobile genetic elements (MGEs), including integrative binding elements (ICEs) and mobile genomic islands (MGIs), poses new challenges in clinical management for *M*. *morganii* [20–22]. Resistance genes were mainly plasmid mediated and harboured by various transposons or integrons, which have significantly contributed to the increased levels of resistance in *M*. *morganii* [23].

Directly targeting defensive mechanisms, therefore, has the potential to greatly improve treatment efficacy when performed in combination with antibiotics or other bactericidal treatments [24]. Thus, understanding bacterial defenses is critical for developing new therapeutics and minimizing the emergence of resistance.

Understanding the genomic signature of *M*. *morganii* will facilitate the identification of unpretentious contrasts within the genome and pathogenicity characteristics. Here in this article, we describe the genomic diversities, Antimicrobial Resistance genes (ARGs), virulence factors and bacterial defense mechanisms found in *Morganella morganii* isolated from a UTI patient in Bangladesh. To understand the underlying factors defining the genetic diversity and molecular epidemiology, through different gene transfer mechanisms and bacterial own defense systems, high throughput WGS and bioinformatic data analysis is essential.

## Methodology

### Sample collection

The samples were collected as part of a project related to antimicrobial resistance analysis of clinical isolates in Bangladesh from a period to January 2022 to December 2023. The specific bacterial sample was collected from a urinary tract infection (UTI) patient. Verbal consent was taken from all the participating members or their legal guardian for this experiment.

In this study, urine samples were first cultured in Blood Agar Plate and MacConkey agar plate to isolate bacteria from Enterobacteriaceae family which are common causes of UTI. After that, bacterial isolates were cultured in liquid broth (LB) media. A small aliquot (~1ml) of broth was sub-cultured from the patient and then genomic DNA was extracted using Wizard Genomic DNA Purification kit (Promega) according to the manufacturer’s instructions. The quality of gDNA was examined using Quantus Fluorometer (Promega) according to the manufacturer’s instructions.

### Phenotypic characteristics analysis

The antimicrobial susceptibility test (AST) was performed using disc diffusion assay in Mueller Hinton Agar (MHA) plate. The following discs were used for the assay, Gentamicin (GEN) 10μg, Amikacin (AK) 30μg, Nalidxic Acid (NA) 30μg, Ceftazidime (CAZ) 30μg, Ampicillin (AM) 30μg, Ceftriaxone (CRO) 30μg, Azithromycin (AZM) 30μg and Optochin antibiotic. The antimicrobial susceptibility was calculated from comparing the zone diameters with the CLSI AST Guidelines (https://clsi.org/meetings/susceptibility-testing-subcommittees/clsi-and-ast/).

The biofilm assay was performed using Microtiter Dish Biofilm Formation Assay method and ATCC strain of *Salmonella enterica* (ATCC 14028) was used as control. Hemolysis activity was checked using blood agar plate.

### Library preparation and whole genome sequencing

Whole genome sequencing (WGS) was performed for research purposes at the Genomic Research Laboratory, BCSIR, Dhaka, Bangladesh. The library preparation of the sample was done using the Nextera ™ DNA Flex Library Preparation kit following the manufacturer’s instructions (Illumina Inc., San Diego, CA). The 300 ng gDNA of the sample was used to prepare paired-end libraries with the Nextera ™ DNA Flex Library Preparation kit with an average insert size of 600 bp for the sample according to the manufacturer’s instructions (Illumina Inc., San Diego, CA). The library was sequenced using a MiniSeq sequencing system (Illumina). The output binary base call (BCL) files were converted to Fastq file format using Illumina bcl2fastq conversion software (v2.20). Fastq files were demultiplexed to single-sample FASTQ files against the sample id using the command line followed by the Illumina bcl2fastq Conversion software (v2.20), software guide.

### Genome assembly, annotation and comparative analysis

The raw reads of the genomes were analyzed by FastQC [25]. To remove low-quality reads, Trimmomatic (v. 0.36) was used [26]. The genome of the bacteria was assembled using SPAdes genome assembler v3.15.5 [27].

To confirm the bacterial species of the isolated strain KmerFinder (v3.2) [28] was used as well as a phylogenetic tree was constructed using the PATRIC platform [29]. The closest reference and representative genomes were identified by Mash/MinHash [30]. PATRIC global protein families (PGFams) [31] was selected from these genomes to determine the phylogenetic placement of this genome. MUSCLE [32] was used for aligning the protein sequences and the nucleotides were then mapped to the protein alignment.

To gain insight into genomic epidemiological relatedness of the isolate with the other circulating strains, a phylogenetic tree was constructed with 67 complete genomes available from different countries including the reference strain, *Morganella morganii* strain MGYG-HGUT-02512 using PARTRIC Platform. Out of those 67 genomes, 42 were from Asia (including our genome), 18 from Europe and 6 from North America.

The *Morganella morganii* genome was annotated using the RAST tool kit (RASTtk) [33]. Subsystem analysis was performed using the PATRIC web server. Furthermore, secondary metabolite analysis was performed using the antiSMASH web server [34].

### Genomic island and Prokaryotic Antiviral Defense mechanism identification

Usually, some regions of bacterial genomes undergo the process of horizontal transfer from different organisms which are known as Genomic Islands (GIs). Numerous significant acquired adaptations of the bacteria that have a significant impact on their evolution and behaviour are frequently a result of these locations. These sites are also important for the rise of their pathogenic and virulent genes. The Genomic Islands of the isolated bacterium were identified using the IslandViewer platform [35].

Furthermore, mobile genetic elements were identified using MobileElementFinder [36]. It identifies mobile genetic elements and their relation to antimicrobial resistance genes and virulence factors. Prokaryotic Antiviral Defense mechanisms were also identified using PADLoc [37]. In this system, CRISPR regions were also identified using CRISPRDetect [38].

### Analysis of antimicrobial resistant genes and virulence genes

Analysis of AMR gene was performed using four different AMR databases and/or bioinformatic tools, namely: AMRfinder [39], CARD [40], NCBI, and ResFinder [41]. These platforms assign to each AMR gene functional annotation, broad mechanism of antibiotic resistance, drug class and, in some cases, specific antibiotic it confers resistance to. Finally, the data derived from all the platforms were compared to portray the real scenario of AMR in the bacterial isolate. Virulence genes were identified using the VFDB analyzer [42].

## Results

### Phenotypic characteristics

The isolate showed rod shaped single colony in nutrient agar plate and showed resistance against Ampicillin, Ceftazidime and Ceftriaxone antibiotic according to CLSI guidelines. It also showed more biofilm in comparison with the ATCC strain in nutrient broth. However, the isolate showed gamma hemolysis i.e., no change in the blood agar media occurred. All the relevant Figs are added in S2 File.

### Genome assembly and comparative analysis

The assembled genome of *M*. *morganii* had 33 contigs, with a total length of 3,730,625 bp and an average GC content of 51.15% (Table 1).

**Table 1:** Assembly details.

Contigs	33
GC Content	51.15%
Plasmids	0
Contig L50	2
Genome Length	3,730,625 bp
Contig N50	1,030,099 bp

This genome is in the superkingdom bacteria and was annotated using genetic code 11 in RAST tool kit (RASTtk). The taxonomy of this genome is:

Cellular organisms > Bacteria > Proteobacteria > Gammaproteobacteria > Enterobacterales > Morganellaceae > *Morganella* > *Morganella morganii*

**Fig 1:**
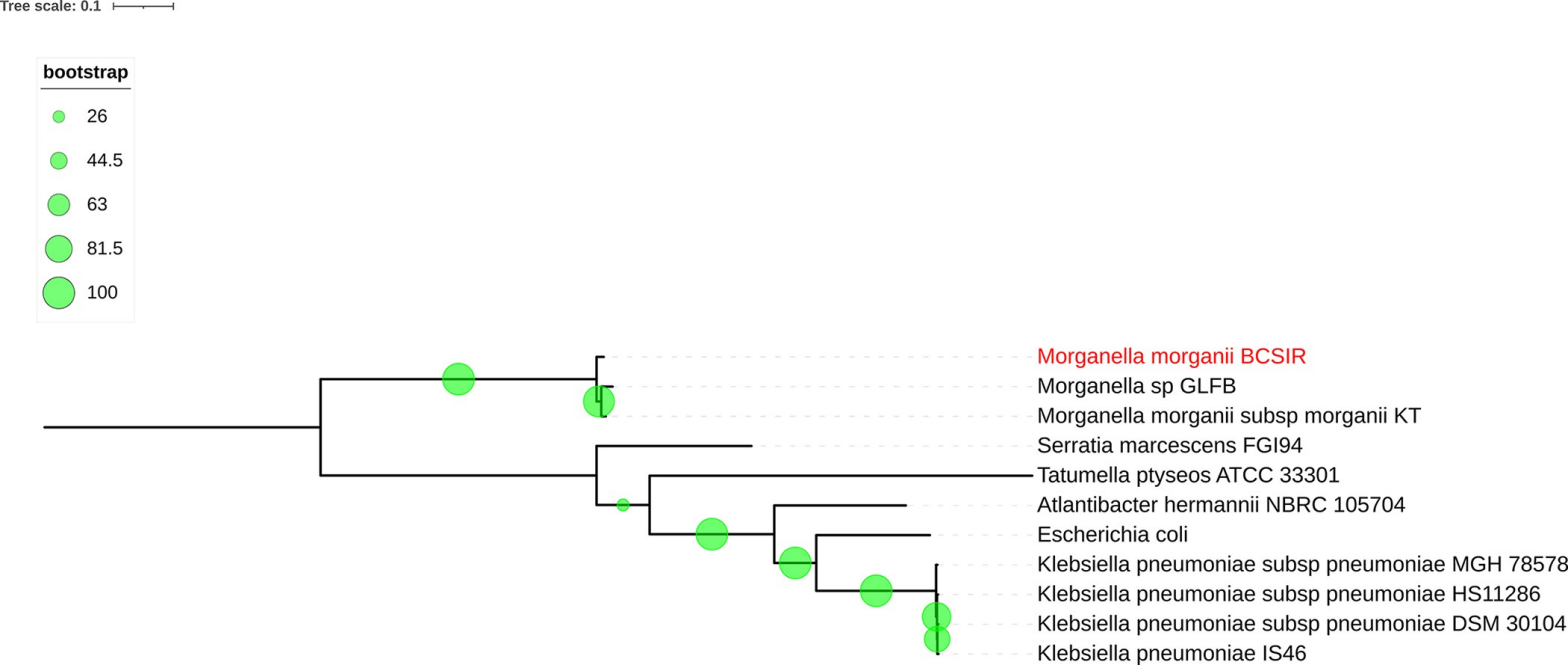
Phylogenetic analysis of the isolated genome for species identification.

The constructed phylogenetic tree (Fig 1) also confirmed the bacterial isolate to be *Morganella morganii*. The isolate was rooted from the same position as the *Morganella morganii* subsp morganii KT genome (GCF_000286435.2).

**Fig 2:**
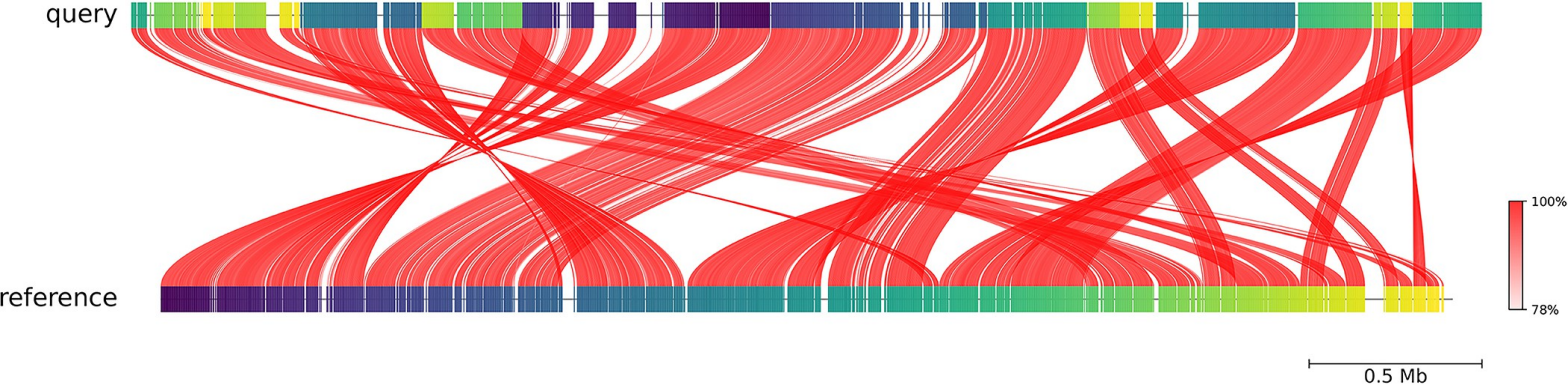
Average Nucleotide Identity (ANI) showing similarity of the isolate with the reference genome.

Average Nucleotide Identity (ANI) calculated against the *M*. *morganii* GCA_902387845.1 showed more than 97% similarity (Fig 2).

**Fig 3:**
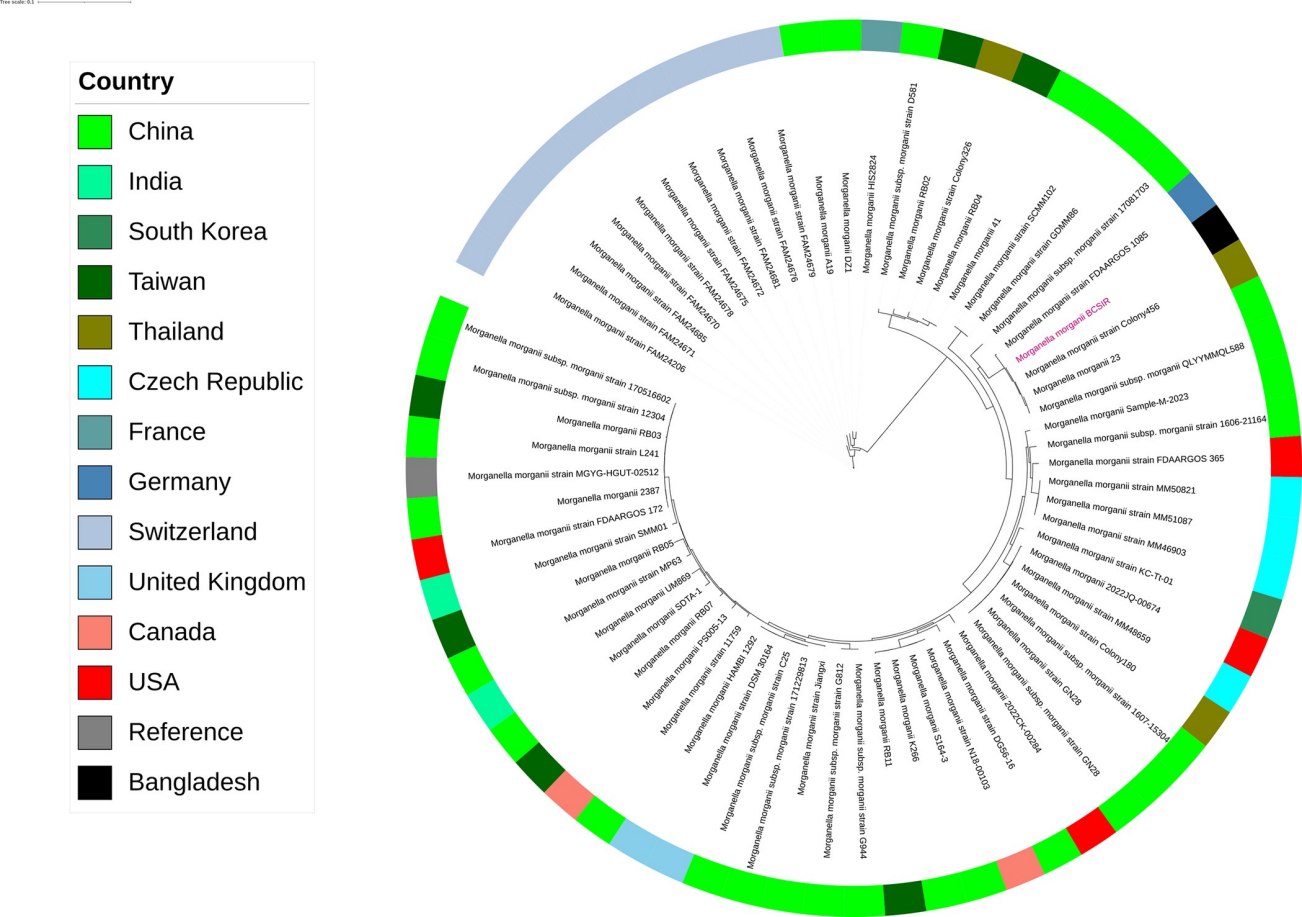
Comparative analysis of the isolated genome with 66 other *Morganella morganii* genomes.

The comparative analysis revealed a close phylogenetic relationship between the isolated Bangladeshi *M*. *morganii* and those previously identified strains in Thailand, China, and Germany (Fig 3).

### Genome annotation

This genome has 3,718 protein-coding sequences (CDS), 73 transfer RNA (tRNA) genes, and 7 ribosomal RNA (rRNA) genes. The annotated features are summarized in Table 2.

**Table 2:** Annotated genome and protein features.

Genome annotation features		Protein Features	
CDS	3,718	Hypothetical proteins	810
tRNA	73	Proteins with functional assignments	2,908
rRNA	7	Proteins with EC number assignments	965
Partial CDS	0	Proteins with GO assignments	776
Miscellaneous RNA	0	Proteins with Pathway assignments	679
Repeat Regions	0	Proteins with PATRIC genus-specific family (PLfam) assignments	3,485

The annotation included 810 hypothetical proteins and 2,908 proteins with functional assignments (Table 2). The proteins with functional assignments included 965 proteins with Enzyme Commission (EC) numbers [43], 776 with Gene Ontology (GO) assignments [44], and 679 proteins that were mapped to KEGG pathways [45]. PATRIC annotation includes two types of protein families, and this genome has 3,485 proteins that belong to the genus-specific protein families (PLFams) and 3,535 proteins that belong to the cross-genus protein families (PGFams).

**Fig 4:**
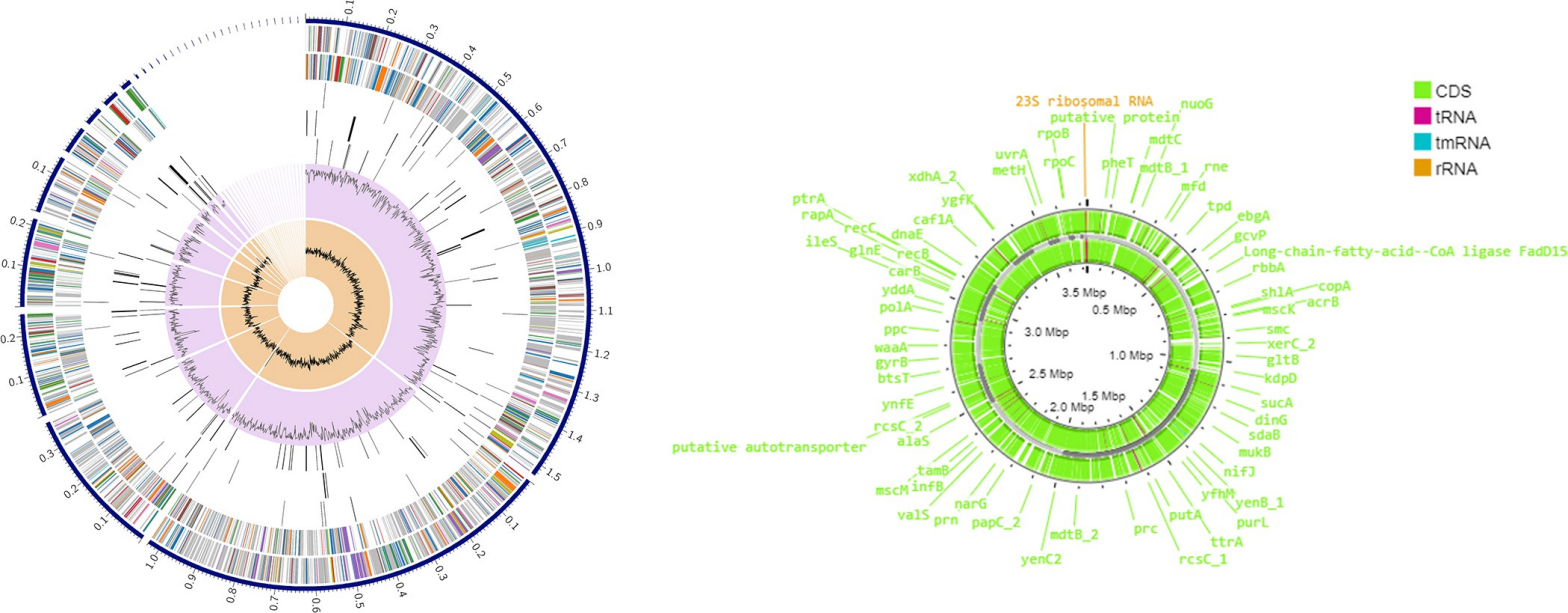
Graphical display of the distribution of the genome annotations.

The distribution of the genome annotations is shown in a circular graphical format (Fig 4A). This comprises the contigs, CDS on the forward and reverse strands, RNA genes, CDS with similarity to known virulence factors and antimicrobial resistance genes, GC content, and GC skew, in order from the outer to the inner rings. The subsystem to which these genes belong is indicated by the colours of the CDS on the forward and reverse strands. The annotation features from the prokaryotic genome annotation tool, Prokka, are shown in Fig 4B. This includes CDs, tRNA, tmRNA and rRNA.

### Subsystem analysis

**Fig 5:**
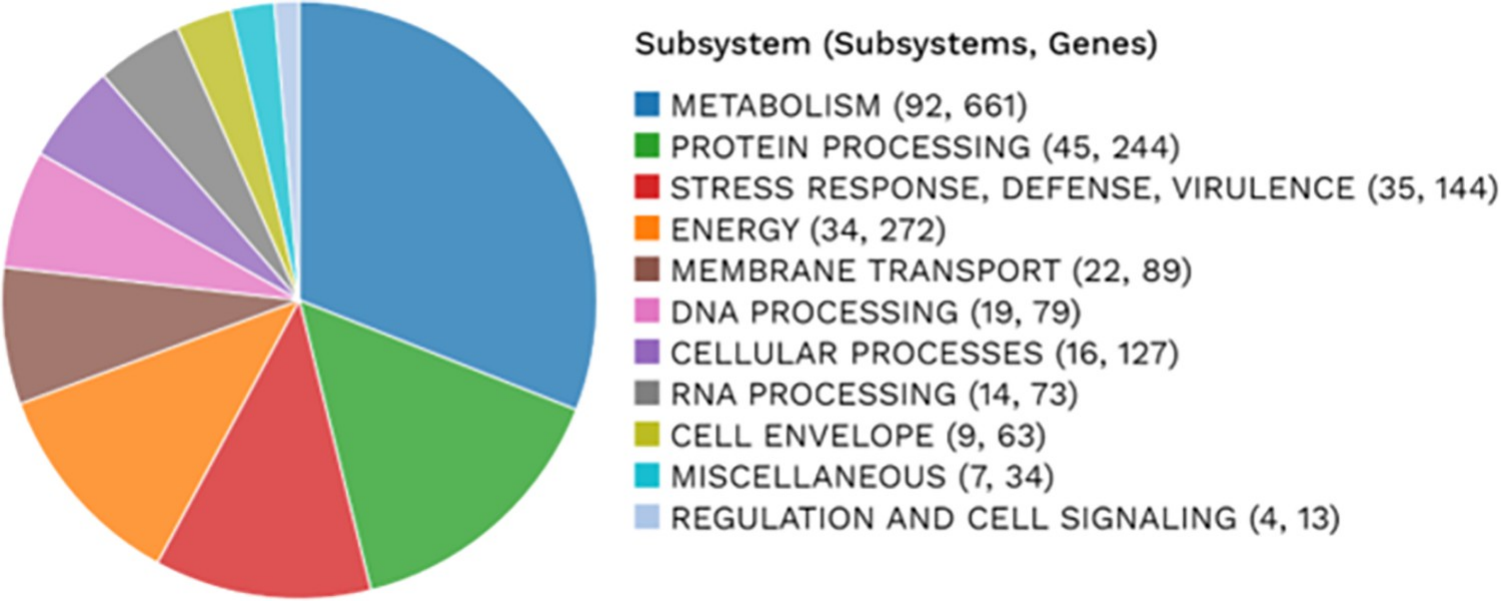
Subsystem analysis showing genes and feature counts found in the isolate.

A subsystem is a set of proteins that together implement a specific biological process or structural complex [46] and PATRIC annotation includes an analysis of the subsystems unique to each genome. An overview of the subsystems for this genome is provided in Fig 5. The analysis here shows valuable information on the functional profile of the bacterial isolate. Most of the genes here are responsible for metabolism, protein processing, stress response, energy and membrane transport, respectively. Metabolism related genes are very crucial for the survival of bacteria by helping in nutrient acquisition, utilization, and energy production.

### Biosynthetic gene cluster analysis

**Fig 6:**
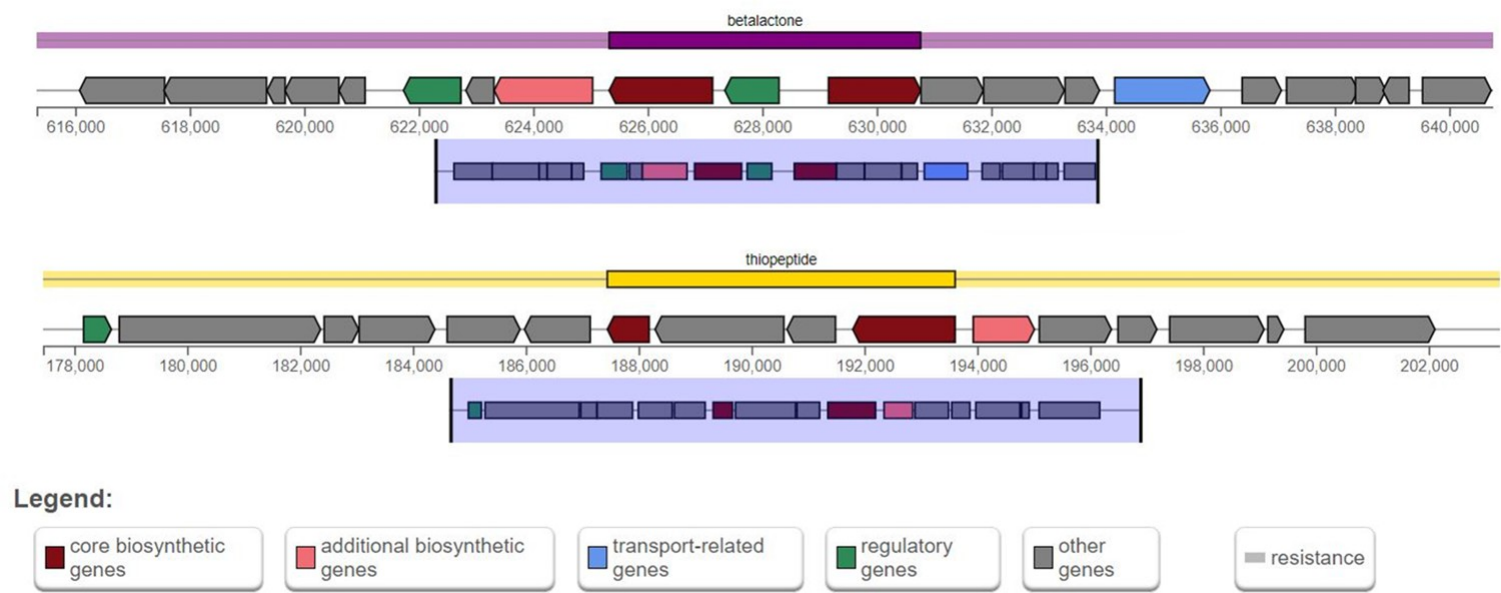
Available biosynthetic gene clusters in the genome.

The analysis of the biosynthetic gene clusters in the isolate has revealed the presence of several significant clusters, including betalactone, thiopeptide, RRE-containing, and RiPP-like peptide clusters (Fig 6). This findings emphasize the isolate’s ability to synthesize a wide variety of bioactive substances. These two essential metabolic compounds (thiopeptides and RiPP-like peptides) have antimicrobial properties.

### Genomic islands of the bacterial isolate

Many genomic islands are flanked by repeat structures and carry fragments of other mobile and accessory genetic elements, such as bacteriophages, plasmids and insertion sequence (IS) elements. These sequences carry the signature of horizontal gene transfer between bacterial populations.

**Fig 7:**
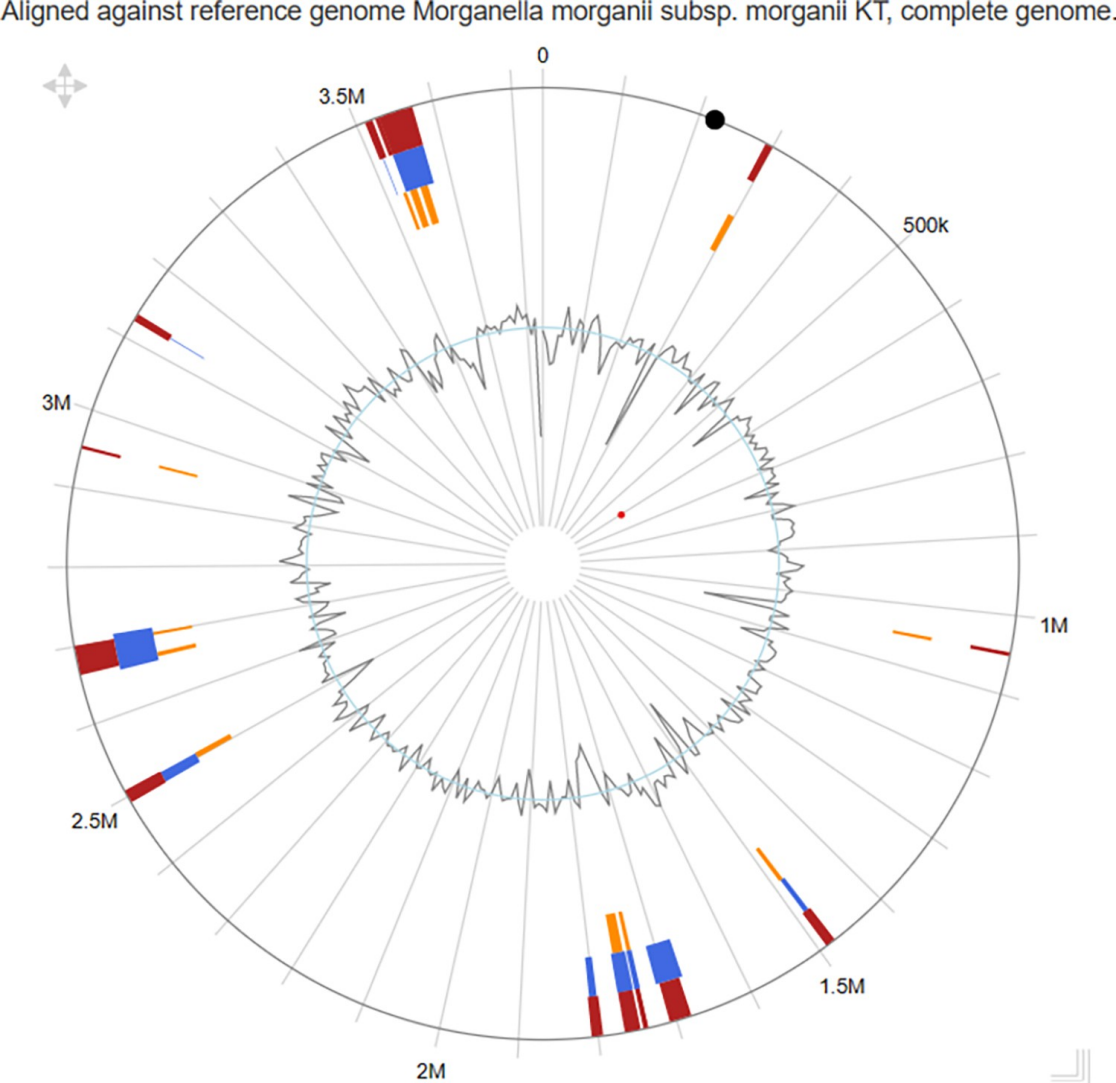
Genomic islands in the bacterial isolate. The red, blue and orange colors show different prediction methods such as Integrated, IslandPath-DIMOB, and SIGI-HMM respectively. The zigzag lines demonstrate the contig boundary in the genome.

To analyze the genomic island of the isolate, several prediction methods were employed, namely Integrated, IslandPath-DIMOB, and SIGI-HMM. The investigation revealed the presence of various phage associated integrase and transposase proteins, along with flagellar biosynthesis proteins (Fig 7). Additionally, the genomic island harboured genes encoding arsenic metallochaperone and nickel ABC transporter. Notably, different toxin proteins, including YpjF toxin protein and Colicin E2 tolerance protein, were also identified in the genomic island. Furthermore, a 43944bp long Type I restriction-modification system was found in the isolate in all the applied methods. Few heat shock proteins and stress proteins were also visible. All the detailed information on the genomic island analysis is added in S1 File.

### Antimicrobial resistance genes and virulence genes

Four different platforms were used to identify the AMR genes present in the isolate. All four databases showed the presence of Beta-lactam resistant gene blaDHA with 100% coverage. The annotated AMR in this genome and corresponding drug resistance is provided in Table 3.

**Table 3:** Antimicrobial resistance genes.

Name of the database/tools	Gene	% Coverage	Resistance to drug
**AMRfinder**	blaDHA	100	
**CARD**	DHA-18	100	cephalosporin; cephamycin
	CRP	100	fluoroquinolone; macrolide; penam
**NCBI**	blaDHA-18	100	Cephalosporin
**Resfinder**	blaDHA-18_1	100	Amoxicillin; Amoxicillin+Clavulanic_acid; Ampicillin; Ampicillin+Clavulanic_acid; Cefotaxime; Cefoxitin; Ceftazidime; Piperacillin; Piperacillin+Tazobactam; Ticarcillin; Ticarcillin+Clavulanic_acid

Two virulence genes kdsA and cheY were found in the isolate after VFDB analysis (Table 4). The kdsA gene encodes for 2-keto-3-deoxy-D-manno-octulosonate-8-phosphate synthase, an enzyme that is involved in the biosynthesis of lipopolysaccharide (LPS), a major component of the outer membrane of Gram-negative bacteria. LPS is an endotoxin with high virulence properties, it also contributes to the ability of bacteria to cause disease.

**Table 4:** List of virulence genes found in the isolate.

Source	Gene	Product	Identity	E-value
VFDB	kdsA	2-Keto-3-deoxy-D-manno-octulosonate-8-phosphate synthase (EC 2.5.1.55)	80	1e-131
VFDB	cheY	Chemotaxis regulator—transmits chemoreceptor signals to flagellar motor components CheY	83	5e-55

The cheY gene encodes for a chemotaxis regulator, a protein that is involved in the process of transmitting chemoreceptor signals to flagellar motor components. It helps bacteria in the movement towards or away from chemical signals. Chemotaxis is important for bacteria to find food and/or avoid predators. It also helps in biofilm formation.

The identification of the kdsA and cheY genes within an isolate implies a potential virulent nature of the organism. This is because LPS and chemotaxis are both important virulence factors.

### Mobile genetic elements

Mobile genetic element finder results showed the presence of two insertion sequences in the genome (Table 5). Both segments have more than 90% identity and good coverage as well. These segments may have been transferred to the isolate through horizontal gene transfer.

**Table 5:** Mobile genetic elements in the isolate.

mge name	type	allele_len	e_value	identity	coverage	gaps	start	stop
**ISEhe3**	Insertion sequence	1229	0	0.921951	0.998373	2	625083	626311
**ISEcl10**	Insertion sequence	1207	0	0.946976	1	0	281172	282378

### Prokaryotic Antiviral Defense mechanisms

**Fig 8:**
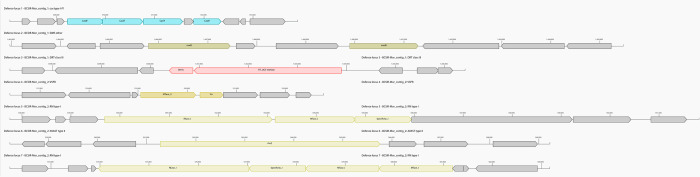
Antiviral defense mechanism in the isolate.

The PADLOC analysis of the isolate’s genome revealed several antiviral defense mechanisms (Fig 8, Table 6). A CRISPR (Clustered Regularly Interspaced Short Palindromic Repeats) region with four Cas genes was identified, which is one of the significant defense systems. In order to guard against subsequent infections of the same virus, bacteria use CRISPR regions to store DNA sequences from foreign viruses.

**Table 6:** Detected defense systems in the genome.

System	Protein name	start	end	strand
cas_type_I-F1	Cas6f	410985	411542	-
cas_type_I-F1	Cas7f	411769	412563	-
cas_type_I-F1	Cas5f	412577	413398	-
cas_type_I-F1	Cas8f	413388	414113	-
DRT_class_III	RT_UG5-nitrilase	309524	312529	+
DRT_class_III	Drt1b	312557	313039	+
RM_type_II	MTase_II	248699	249814	+
RM_type_II	REase_II	249901	250371	+
RM_type_I	REase_I	269328	272741	+
RM_type_I	MTase_I	272805	274439	+
RM_type_I	Specificity_I	274436	275584	+
AVAST_type_II	Avs2	290922	295397	+
RM_type_I	REase_I	303613	306675	-
RM_type_I	Specificity_I	306679	307836	-
RM_type_I	MTase_I	307837	309327	-
RM_type_I	MTase_I	309333	310832	-

The restriction-modification (R-M) system is another crucial defense mechanism in bacteria, and it was found in the isolate. By identifying and cleaving particular DNA sequences, R-M systems can stop viruses from invading cells. These antiviral defense mechanisms for bacteria are among the oldest and most common. Two major types of R-M systems include MTase-type and REase-type.

## Discussion

With the advancement of whole genome sequencing technologies, the genomic analysis of the pathogenic clinical bacterial isolates has become more advanced and sophisticated. This is opening new horizons in finding out more about such pathogens. A prevalent but largely disregarded environmental opportunistic pathogen that can lead to fatal nosocomial infections is *Morganella morganii*.

Here in this study, whole genome sequencing was performed in a clinical isolate of *M*. *morganii* from a Bangladeshi patient. Detailed analysis of the sequencing data revealed some exciting pathogenic features of the isolate and how these genes spread among bacterial population.

The de novo assembly showed 51.15% GC content of the isolate which is similar to the amount found in other publications as well [2, 47]. Additionally, phylogenetic analysis was performed to confirm the identity of the isolate which revealed that the isolate is an opportunistic bacteria *M*. *morganii*. More than 97% similarity was found in the Average Nucleotide Identity (ANI) comparison to the reference genome of *M*. *morganii*, GCA_902387845.1 whereas ANI values less than 95% signifies differences in strains [48, 49].

The genome annotation results showed 3718 CDS and 2908 proteins with functional assignments. With the exponential growth of genome sequencing of microbial population functional annotations require more automation like subsystem analysis for reliable results [46]. Here, the subsystem analysis identified several metabolic genes as well as virulence and defense-related genes. According to the gene numbers, mostly metabolism, protein processing, stress response, defence, virulence-related genes were mostly prominent. Interestingly, pathogenic bacteria acquire genes encoding metabolic functions during the evolutionary process to have a selective advantage in host environments [50].

Secondary metabolites produced by biosynthetic gene clusters of bacteria show significant importance in inspecting the valuable novel biomolecules like antibiotics, pigments, growth hormones etc. [51]. Here, the analysis showed the presence of betalactone, thiopeptide, RRE-containing, and RiPP-like peptide clusters in the genome of isolated *M*. *morganii*. Betalactone and thiopeptide are often produced in response to stress and/or environmental changes, while the most widely used class of antibiotics is Betalactone [51]. All these metabolites show antibiotic and antifungal properties whereas RRE-containing and RiPP-like peptides show anti-inflammatory activity [52].

Genomic Islands are usually a cluster of genes that give new functionalities, like antibiotic resistance, virulence, and others, and they are frequently horizontally transferred between bacteria [53]. In this study, several phage-associated transposase and integrase were found which signifies the horizontal gene transfer using these segments. Mobile genetic element finder analysis also revealed the presence of two insertion sequences. These insertion sequences can be transferred to bacteria during the infection of phage or transduction methods [54]. Moreover, several metal transporter genes such as arsenic metallochaperone and nickel ABC transporter signify the common presence of *M*. *morganii* in environmental sources. However, the presence of restriction modification systems, stress proteins, and toxin proteins tells the story of how the bacterium survived the evolutionary process by taking up such virulence and defense genes.

To further elucidate the antimicrobial resistance and virulence factors, some specific bioinformatics tools were utilized. The results showed Beta-lactam resistance with the presence of the blaDHA gene in the genome and phenotypic resistance profile from antimicrobial susceptibility test. Combining the results from four different databases emphasizes the confidence of the outcome. Moreover, since the isolate didn’t have any plasmid in its genome, it shows that the resistance gene is located in the chromosomal DNA. Furthermore, two virulence genes kdsA and cheY were found in the VFDB analysis. Both of them show high virulence properties in gram-negative bacteria by regulating LPS biosynthesis, bacterial chemotaxis and biofilm formation [55, 56].

However, bacteria have their own defense mechanism to protect themselves from phages and other foreign attacks. Among them CRISPR-Cas systems and restriction modification systems are the most common form of defense in bacteria [57]. In this study, PADLOC analysis revealed the presence of a CRISPR region in contig 1 with four Cas type I-F genes. Other reports also showed the presence of type I-F Cas genes in *Enterobacteriaceae* [58]. Moreover, two types of restriction modification systems were also found in the genome of the isolated *M*. *morganii*. Methyltransferases (MTases) are used in MTase-type R-M systems to modify particular DNA regions. The restriction enzyme recognizes these methylation regions as self, which stops it from cleaving the DNA. Whereas a restriction enzyme (REase) is used in R-M systems of the REase type to cleave particular DNA sequences. Only unmethylated DNA can be attacked by REase.

These antiviral defense systems may indicate that the isolate is well-defended against viral infection. This is crucial for the isolate’s capacity to endure and flourish in its surroundings.

Both restriction modification systems and CRISPR work directly on cleaving DNA sequences in different mechanisms [59] and protect against the invasion of foreign DNA.

This study focuses on the potential trade-off between the available defense systems against phages and the acquisition of virulence and resistant genes. The identification of such genes in the genome of the isolated strain may help guide the development of targeted therapies and the identification of potential drug targets. Additionally, the findings highlight the importance of understanding the defense mechanisms of these pathogenic bacteria in healthcare settings, as well as the need for continued surveillance of antibiotic resistance in clinical isolates.

## Supporting information

S1 File Details of genomic island analysis. (XLSX)

S2 File Results of AST, hemolytic activity test and biofilm assay analysis. (DOCX)
