# Supplementary material for: An insight into the genome-wide analysis of bacterial defense mechanisms in a uropathogenic Morganella morganii isolate from Bangladesh
Source: PLoS One. 2025 Jan 23;20(1):e0313141. doi: 10.1371/journal.pone.0313141 (PMC11756799; doi:10.1371/journal.pone.0313141)
Supplement: S2 File — (DOCX) [file pone.0313141.s002.docx]

**Supplementary file 2**


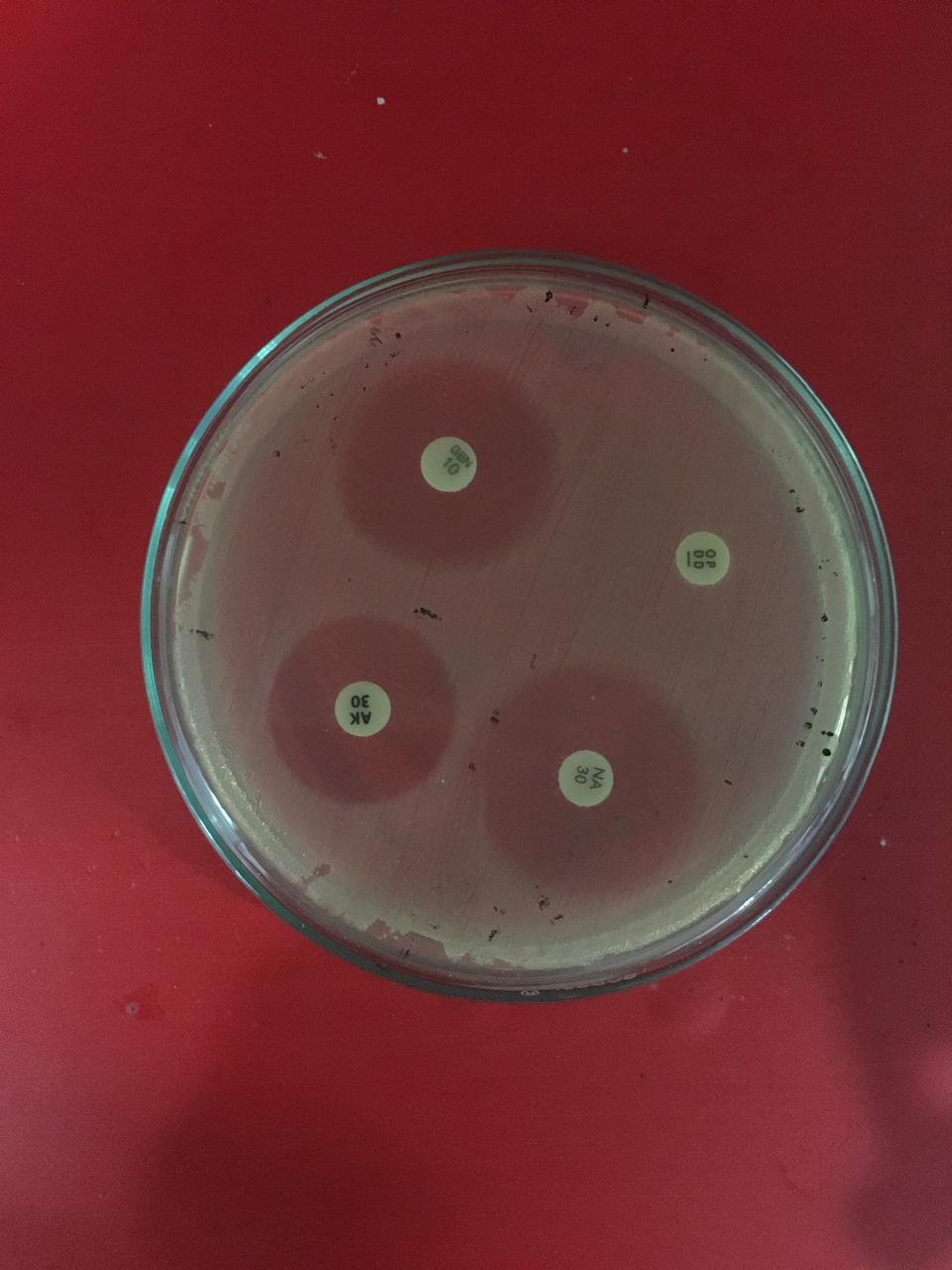

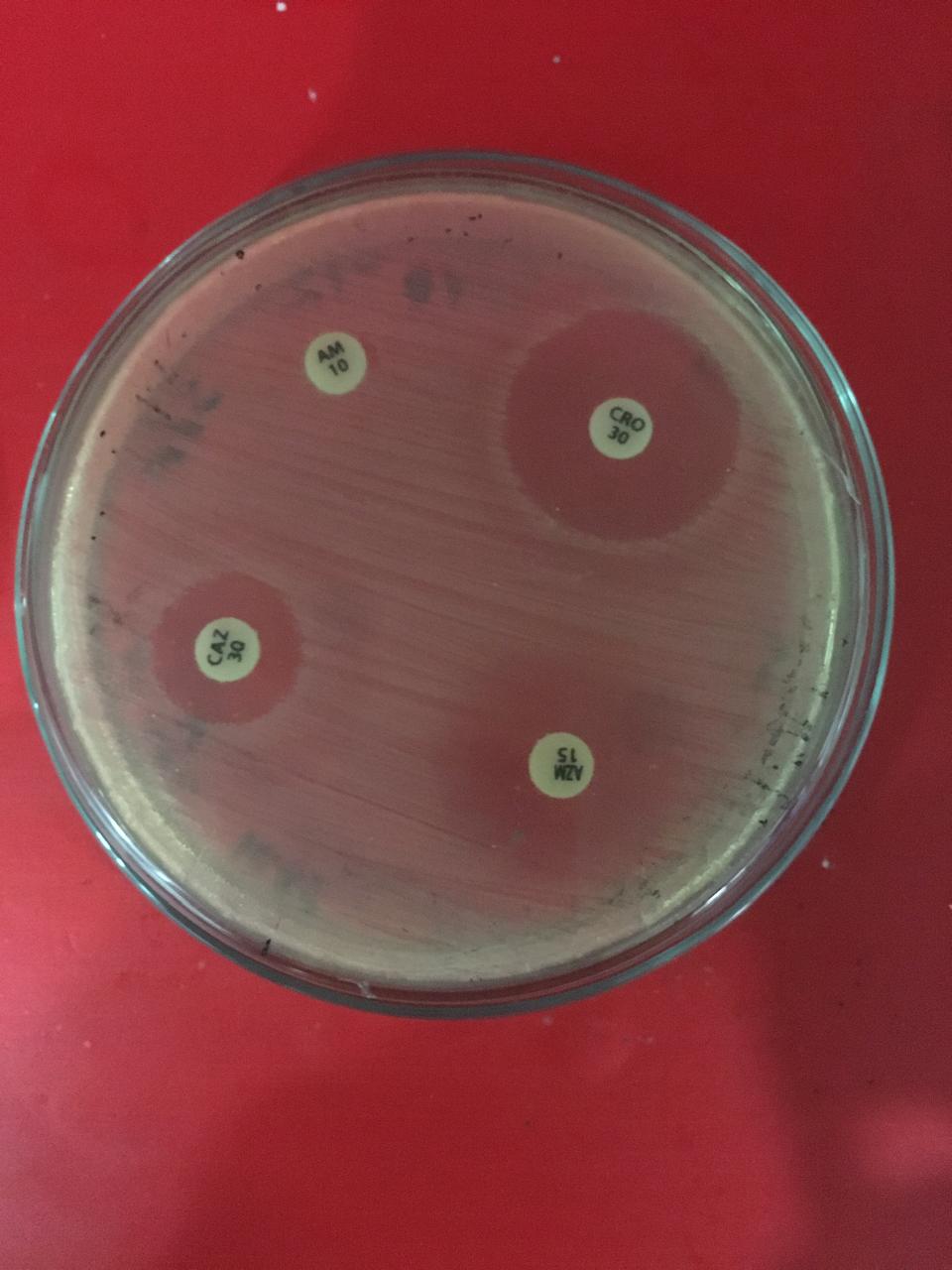


S1 Fig.: Antimicrobial Susceptibility Test (AST) of the isolate using disc diffusion method. Two different MHA plate was used for better zone interpretation


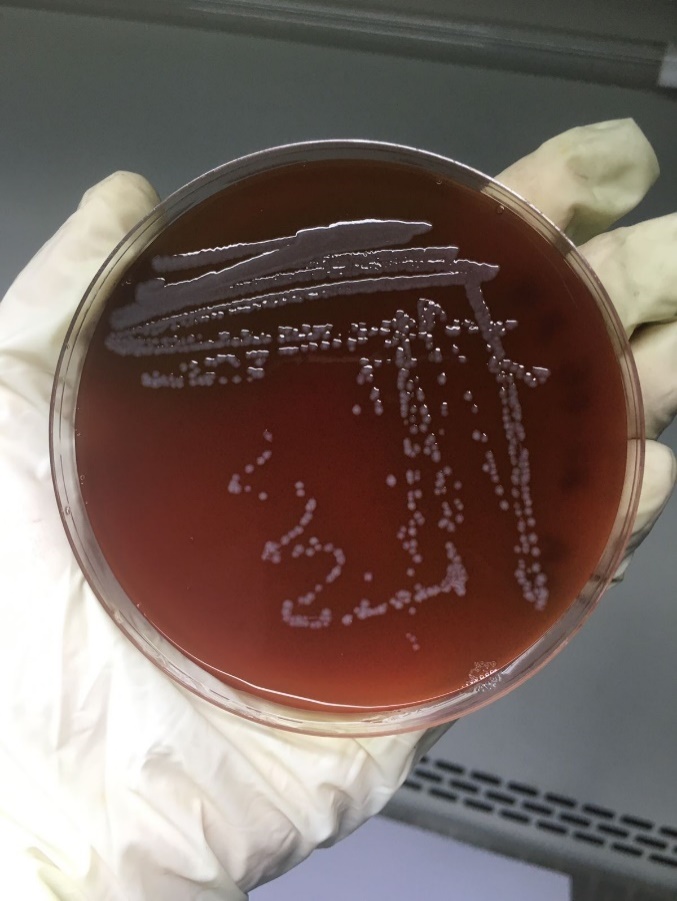


S2 Fig.: *Morganella morganii* isolate showing no hemolytic activity in blood agar plate

S1 Table: Biofilm assay of *M. morganii*. The wavelength is measured in 590nm.

| Set 1 | *M. morganii* | ATCC 14028 | Blank |
| --- | --- | --- | --- |
| Replication 1 | 0.328 | 0.176 | 0.113 |
| Replication 2 | 0.299 | 0.187 | 0.113 |
| Replication 3 | 0.308 | 0.179 | 0.122 |
